# Exploring the space for task shifting to support nursing on neonatal wards in Kenyan public hospitals

**DOI:** 10.1186/s12960-019-0352-x

**Published:** 2019-03-06

**Authors:** Jacinta Nzinga, Jacob McKnight, Joyline Jepkosgei, Mike English

**Affiliations:** 1 Health services and Research Group, Kenya Medical Research Institute/Wellcome Trust Research Programme, PO Box 43640, Nairobi, 00100 Kenya; 2 Nuffield Department of Medicine and Department of Paediatrics, University of Oxford, Oxford, United Kingdom

**Keywords:** Task shifting, Task sharing, Delegation, Neonatal nursing, Low- and middle-income countries (LMICs), Subconscious triage, Routines, Supervision

## Abstract

**Background:**

Nursing practice is a key driver of quality care and can influence newborn health outcomes where nurses are the primary care givers to this highly dependent group. However, in sub-Saharan Africa, nursing work environments are characterized by heavy workloads, insufficient staffing and regular medical emergencies, which compromise the ability of nurses to provide quality care. Task shifting has been promoted as one strategy for making efficient use of human resources and addressing these issues.

**Aims and objectives:**

We aimed to understand the nature and practice of neonatal nursing in public hospitals in Nairobi so as to determine what prospect there might be for relieving pressure by shifting nurses’ work to others.

**Methods:**

This paper is based on an 18-month qualitative study of three newborn units of three public hospitals—all located in Nairobi county—using an ethnographic approach. We draw upon a mix of 32 interviews, over 250 h’ observations, field notes and informal conversations. Data were collected from senior nursing experts in newborn nursing, neonatal nurse in-charges, neonatal nurses, nursing students and support staff.

**Results:**

To cope with difficult work conditions characterized by resource challenges and competing priorities, nurses have developed a ritualized schedule and a form of ‘subconscious triage’. Informal, organic task shifting was already taking place whereby particular nursing tasks were delegated to students, mothers and support staff, often without any structured supervision. Despite this practice, nurses were agnostic about formal institutionalization of task shifting due to concerns around professional boundaries and the practicality of integrating a new cadre into an already stressed health system.

**Conclusion:**

Our findings revealed a routine template of neonatal nursing work which nurses used to control unpredictability. We found that this model of nursing encouraged delegation of less technical tasks to subordinates, parents and other staff through the process of ‘subconscious triage’. The rich insights we gained from this organic form of task shifting can inform more formal task-shifting projects as they seek to identify tasks most easily delegated, and how best to support and work with busy nurses.

**Electronic supplementary material:**

The online version of this article (10.1186/s12960-019-0352-x) contains supplementary material, which is available to authorized users.

## Background

Sustainable Development Goal 3.2 calls for a reduction in neonatal mortality to at least 12 per 1 000 live births [1]. High impact, low cost interventions could avert more than 71% of neonatal deaths, but depend to a large extent on effective coverage with facility-based care [2]. However, the performance of public sector hospitals in low- and middle-income countries (LMICs) is often poor [3, 4], and this is particularly the case for newborn care [5, 6]. Some of the contributing factors are inadequate material resources and equipment, poor adherence to evidence-based guidelines, inadequate human resources for health (HRH) and poor management of material and human resources [6–8].

Nurses are key to the provision of quality care and have a particular influence on newborn health outcomes in hospitals as primary care givers to this highly dependent group [9]. However, according to the Global Health Workforce Alliance (GHWA) 2014 report ‘A Universal Truth’, the global shortage of health workers is estimated to be over seven million [10]. Kenya has an acute shortage of nurses in the public sector, with densities ranging between 1.2 and 0.008 per 1 000 population across counties compared to a new suggested minimum health workforce threshold of 4.45/1000 population for doctors, nurses and midwives combined [11]. Nairobi county, the specific focus of our study, suffers from both major shortages of nurses providing frontline neonatal care in the public sector and very poor neonatal outcomes [12]. Indeed, in Nairobi’s public hospitals, recent work suggests nurse: baby ratios of 1:15 [13]. In countries such as the United Kingdom, even for babies who do not require intensive care, guidelines suggest one nurse for every two to four sick babies [14, 15] with evidence suggesting a relationship between lower nurse ratios and higher mortality [14]. Kenya’s public-sector nursing workforce challenge is however complex. Recent data reveals the country has more than 50 000 nurses registered to practice, but fewer than 17 000 offering care in the public sector, which is particularly relied upon by the poor for inpatient care because of inadequacies in public finance [16]. Workforce solutions must therefore carefully consider budget impacts.

The World Health Organization (WHO) suggests task shifting and sharing as a means to lessen the problems of HRH shortages, while potentially improving access and maintaining or improving quality [17]. ‘Task shifting’ is a phrase used to cover a variety of interventions, but WHO uses it to group activities in which: ‘…trained cadres who do not normally have competencies for specific tasks deliver them and thereby increase levels of health care access’ [18]. The related term ‘task sharing’ is described as ‘the rational distribution of tasks among trained and supervised health professionals and health workers’. In this study, we use the term task shifting although in the context under study tasks that are shifted might still be supervised by a nurse.

In most cases, task shifting and task sharing aim to provide services at reasonable cost through a new cadre of worker when an absolute shortage of staff puts unreasonable demands on existing cadres who are unable to meet requirements [19]. A series of studies and systematic reviews intended to inform WHO’s ‘Recommendations for Optimizing Health Worker Roles to Improve Access to key Maternal and Newborn Health Interventions through Task Shifting’ (OPTIMIZEMNH) (WHO [18]) have reported ‘barriers’ such as training and supervision challenges, problems with professional hierarchies and poor integration of new cadres into formal health systems [20, 21]. Reported facilitators of task shifting included public recognition, creation of visible ties to the formal system where formal health workers were involved in training new cadres [22, 23]. However, Mijovic et al. [24] highlight that in all the African case studies they reviewed, the cadre taking up additional shifted tasks exceeds the formal mandate, taking on responsibilities additional to those ‘shifted’. Hence, while the financial case for task shifting is clear [25], implementation is difficult and the intervention ought not be thought of as a ‘cure-all’.

Task sharing and shifting have been used extensively in LMIC contexts in response to HIV/AIDs and in anaesthetic and surgical care where tasks that are traditionally the preserve of trained physicians are performed by non-physician clinicians or nurses [26]. Task shifting and task sharing is also now common in high-income countries’ hospitals where health care assistants undertake many ‘basic’ nursing tasks under the supervision of nurses [27]. Historically, Kenya had a cadre of non-professional assistants referred to as ‘nurse aides’. Our respondents confirmed that this position was phased out in the early 1990s having been linked to reports nurse aides were over-stepping role boundaries (typically when operating without supervision) and thus putting patients at risk. This might partly explain reluctance to formally re-introduce such a cadre [28]. Anecdotal evidence indicates, however, that shifting of nursing tasks to non-qualified personnel is happening although not officially allowed in the Kenyan public sector.

Our work aimed to explore the potential for task shifting to support the provision of basic nursing care in hospitals’ neonatal units. In 2017, the Kenyan Ministry of Health launched the *Task Sharing Policy 2017–2030* and the *Task Sharing Policy Guidelines* [29]. These policies focused on legitimating task-shifting between existing health professionals by redefining scopes of practice to reflect the realities of routine work and its evolution over more than 20 years (e.g. nurses run primary care, prescribe and put up intravenous fluids in clinical settings). The policy, however, makes no mention of new hospital-based cadres to support basic inpatient nursing care.

Prior task shifting and sharing approaches in LMICs highlight the importance of well-designed interventions linking newly established cadres to existing professionals at the micro level, and their integration into formal structures of health systems [20–22, 30, 31]. Relatedly, De Sardan [32] describes the disconnect resulting from implementing standardized interventions without consideration of everyday contexts. In particular, he describes ‘practical norms’ as ‘the various informal, de facto, tacit or latent norms that underlie the practices of actors, which diverge from the official norms (or social norms)’ ([33]: 26). It is important then to investigate the socio-cultural dimensions or ‘software’ of health systems, including practical norms, as part of any potential task-shifting design efforts [34, 35].

This study aimed to explore the current operation of Nairobi’s New Born Units (NBUs) using an ethnographic approach. It is part of a body of work exploring major gaps in quality of neonatal care and potential solutions, including task shifting. The study reveals nursing routines, highlights areas of working practice that might easily be shared with a lower cadre, describes nursing stakeholders and nurses’ perceptions of task-shifting for this context and explores the overall potential for task shifting in Nairobi’s public hospital NBUs.

## Methodology

This work first started with an exploration of the views of influential nursing leaders on the idea of task shifting as a possible intervention to improve in-patient care for sick newborns. The results of the first phase were complemented by a formal stakeholder analysis [28] and informed empirical work in the second phase of the research. Phase two explored how nursing tasks are performed within the busy, highly normative culture of Kenyan newborn nursing.

### Specific study sites

The second phase was conducted in Nairobi City county, within newborn units of three public hospitals. All hospitals offer inpatient and outpatient services (e.g. immunization, HIV treatment and care and maternity services), and one hospital is specifically a maternity hospital. The hospital descriptions are provided below (Table 1).

**Table 1:** Description of structure and services of the study hospitals and their newborn units (unpublished data)

Hospital	Description					
Hospital code	1		2		3	
NBU bed capacity and no. of deliveries	84 beds/cots with approximately 400 admissions monthly (4 800 yearly)		31 bed/cots combined with approximately 150 admissions monthly		35 beds/cots combined (15 cots) with 100 admissions monthly	
NBU staffing	32 staff		16 staff		17 staff	
	2 paediatricians		2 paediatricians		1 paediatrician	
	6 medical officers		1 medical officer		1 medical officer	
	6 registered clinical officers		0 registered clinical officers		0 registered clinical officers	
	18 nurses		12 nurses		13 nurses	
	2 clinic assistants		1 support staff		2 support staff	
	2 support staff					
Distribution of NBU nurses per shift	Weekdays	Weekends	Weekdays	Weekends	Weekdays	Weekends
	Morning 4	Morning 4	Morning 2–3	Morning 2–3	Morning 2–3	Morning 2
	Afternoon 2	Afternoon 2	Afternoon 2	Afternoon 2	Afternoon 2	Afternoon 1–2
	Night 3	Night 3	Night 1	Night 1	Night 2	Night 2

### Sampling

The first phase of this study involved a total of 10 interviews conducted with senior stakeholders (see Table 2). We then purposively sampled nurses who had significant work experience in the new born unit (our participants’ NBU experience ranges 1–5 years). These included support staff, nurse managers (in-charges) and frontline nurses (junior nurses), but in one setting, we also included nurse students (whose clinical rotations within the NBU typically last 2–6 weeks). To ensure maximum variation of the participants, we included different nursing grades and specialisms that pertain to neonatal nursing cognizant of gender and age variations across our samples. We conducted 22 interviews with hospital staff across all 3 hospitals.

**Table 2:** Study sample size showing number of interviews in each hospital, cadre of health workers and details of stakeholders interviewed

Hospital	Nurses	Support staff	Students	Stakeholders (*n* = 10)
1	6	1		Ministry of Health, Nairobi City County Health Team, Nursing Council of Kenya, National Nurses Association of Kenya, Kenya National Union of Nurses, Kenya Medical Training College, Kenya Paediatric Association and the Kenya Medical Association.
2	8	1	2 FGDs	
3	3	1		
	17	3	2	

Interviews were semi-structured and followed an ethnographic or *long* approach with the aim of not eliciting ‘answers’ but rather invoking narratives [36] and lasted 1–1.5 h. From these narratives, clarifying questions were developed to iteratively check and extend the theory being developed (see interview guide in the Additional file 1). The interviews were complemented by non-participatory observations of day and night shifts, over weekdays and weekends, across all the three hospitals’ neonatal wards (250 h in total).

Interviews were transcribed by one author (JJ), and both transcripts and field notes imported into Nvivo 10 Qualitative Software as a shared project. Three authors (JN, JM and JJ) independently coded data into emerging themes in a first phase of analysis. The team then agreed on a set of themes for the next round of analysis. Initial themes were refined during the research by modifying the interview guide to probe emerging theory, relating it to important questions in the literature on task shifting. In this way, we were able to usefully extend theories on task shifting [37, 38].

We present a full exploration of the ethnographic work elsewhere. Here, we draw on all the interviews and non-participant observation to present a detailed deep description of the organization of nursing work, specifically detailing the conduct of ‘nursing tasks’ and routines as part of inpatient neonatal care. We use the insights gained to explore the potential of task shifting in newborn units. Our findings should not be read as ‘barriers’ and ‘facilitators’ of task shifting but rather as rich description of an environment where task shifting might be implemented. We present findings under two main themes as described below.

## Results

### Organization of neonatal nursing work

**Fig. 1:**
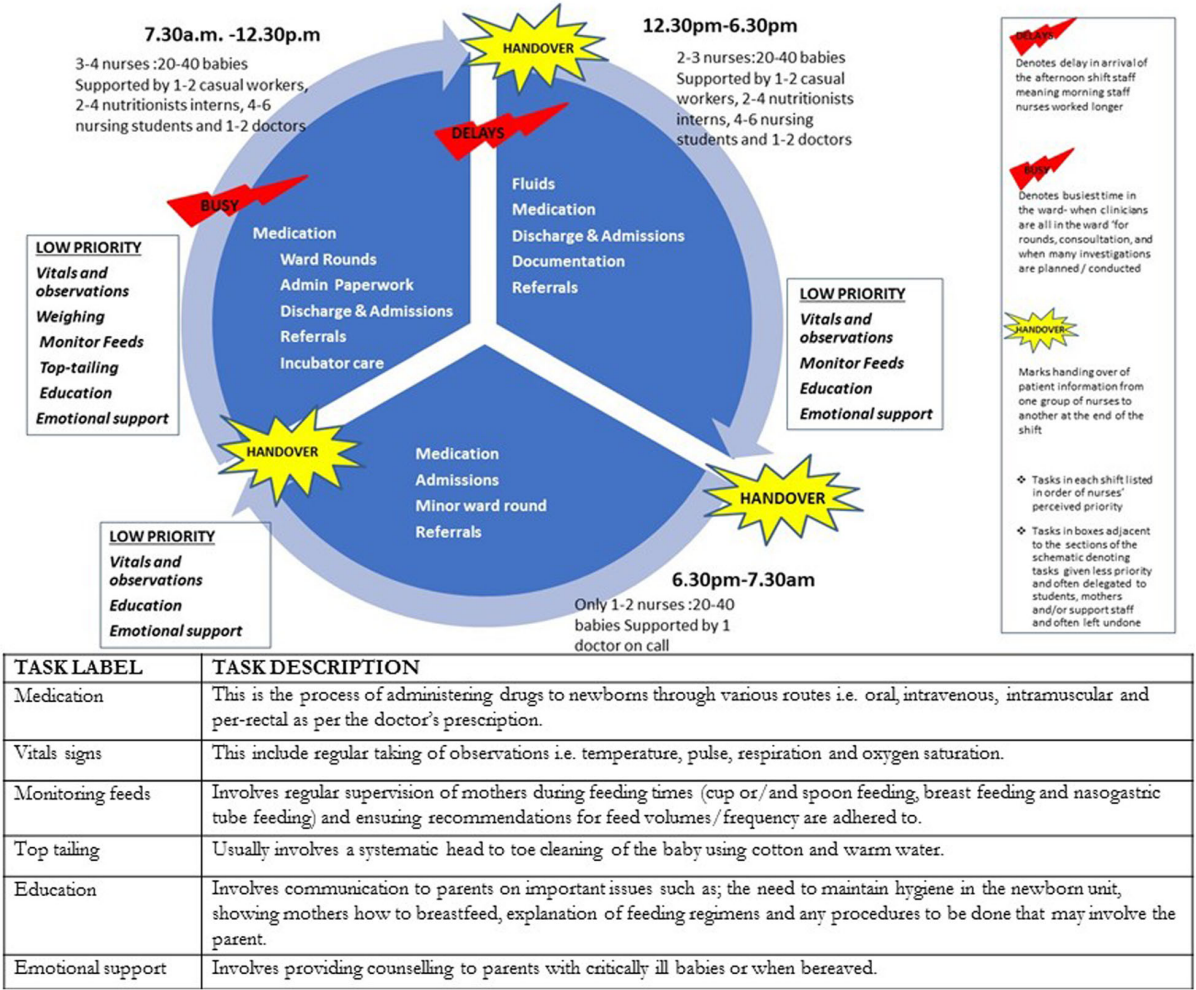
Emerging template of neonatal care in study hospitals depicting how nursing tasks are organized (prioritized tasks in bold, low priority tasks in boxes) against the number of nurses on the three shifts. Of note, ‘Handovers’ with generally very busy morning shifts and delays in staff reporting for the afternoon shift

Nurses, even on these relatively large inpatient newborn units, very rarely have specific post-basic training in neonatal nursing with most being diploma level (registered) nurses who have 3 years general nursing training. In all three hospitals, nurses lacked explicit job descriptions and were not given formal orientation to the neonatal ward setting at the start of work in these units. Thus, nurses’ roles were implicitly drawn from their prior experience and immediate, experiential ‘on-job’ training with everyone expected to fit into roles as depicted in the schedule below (Fig. 1).

It is automatic – nursing is a routine so everyone who is on duty knows what to do in case the in charge is not around it is not new; in every ward you go that is the routine (NBU Nurse Hospital 2)

Across all hospitals, work in the NBU followed a standard routine (see Fig. 1) which was characterized by busy morning shifts where multiple clinicians, nurses, nutritionists and students were present; when care planning was largely conducted; and when most tasks were performed. Afternoon shifts were generally short, but commonly made longer by delays in the arrival of the oncoming shift nurses, and were understood to be less busy than morning and night shifts except when there were emergencies. The longest shift was the night shift, which was least staffed with often one nurse dealing with 15–20 babies over 13 h. Across all three shifts, nursing tasks were performed following a routine that prioritized technical tasks over other bedside care tasks as shown in Fig. 1.

We observed that routines were disrupted by emergency admissions, unexpected staff absences and changing conditions of the very sick babies. In those situations, nurses were forced to make decisions about how to use their time, judging who needed immediate care and who could wait. They did this by using a type of ‘Subconscious Triage’. We use this term to characterize the response to the continual need to make decisions about allocating limited resources while under extreme pressure. We asked nurses what guides them when they have staff shortages:

When you are so tied up [due to shortages], you could start with the very sick one because we do it at 9 a.m. So, because you’re so busy, you do to the very sick ones first. So, make sure they are done at least if the doctors round is being done they need to know the vital signs. So, you do those ones. Then these other ones may not be strictly done at 9. You can even do it at 11, 12 when you are a little relaxed now. So, you can always postpone some and do them later. (NBU Nurse Hospital 1)

On close observation and reflection, we also noticed that nurses followed a common, implicit hierarchy in managing tasks. For example, typically, the provision of bedside care (personal, direct care, e.g. informing mothers of their baby’s condition) only occurred while on the way to perform more technically difficult clinical tasks such as administering intravenous medication or monitoring blood transfusion. Thus, there was tension between the nursing role as providing holistic care versus provision of the more technical, clinical tasks considered ‘crucial’ and often given priority over bedside caring tasks.

Our core thing is the baby, so our priority will be the wellness of the baby, so if I have all that to do there are things I will have to skip, doing resuscitation is a must that one is a must and that is a priority. So, we will have to stabilize the baby with the help of the COs, the clinicians, doctors, until you are sure that the baby is okay and stable. From there now you can do the other bit of it – giving treatment is a must and timings really matter. (NBU Nurse Hospital 2)

Resuscitations were regarded as a priority, but the ‘wellness of the baby’ was generally interpreted as whether or not the baby got all medical treatments and procedures prescribed. This is perhaps inevitable given the pressures that nurses work under and the frequency with which they are expected to deal with more technical, life-threatening issues such as resuscitations.

### Task delegation in practice

The observations above are important because they indicate several areas in which a task-shifting cadre might add value in support of nurses’ work. They also provide essential context that might help design a programme likely to be accepted and supported by nurses. Indeed, we observed ad-hoc shifting and delegation of some tasks across all three NBUs. Our observations of tasks delegated in practice contrasted with those thought safe to delegate by senior nursing stakeholders in related research [39] as did the levels of supervision required for tasks delegated to students (Table 3).

**Table 3:** Task delegation and shifting in practice based on our ward observations

Category of delegation	Task areas
Tasks that can never be delegated to someone other than a qualified professional	Ordering supplies and equipment from the stores and pharmacy Resuscitation of babies Final assessment of nursing students to qualify as nurses Referral of babies from one hospital to an often-higher care hospital
Delegated to student nurses undertaking clinical training attachments	Weighing babies Taking vital signs observations Giving intravenous fluids Filling the nursing Kardex (*a summary of individual patient needs used by nurses to communicate important information on their patient and often updated at every shift change*). Giving treatments—either oral, intra-muscular or intravenous
Delegated to mothers	Cup and NGT (naso-gastric tube) feeding of babies Filling amounts of milk fed to babies on feeding chart Top tailing (cleaning baby from head to toe)
Delegated to support staff^a^	Dusting and cleaning incubators Cup and NGT (naso-gastric tube) feeding of babies Top tailing (cleaning baby from head to toe)
Tasks sometimes done/left undone	Education Emotional support

^a^Long-term casual employees often sub-contracted by the hospital to primarily provide cleaning services within the hospital although they are also often used to run errands within the different departments in the hospital

We observed that it was mainly the non-clinical tasks that were delegated or shifted and that this happened only under authorization (but rarely supervision). The authorization only came from the more senior and experienced nurses in the ward and never the junior nurses.

I: You usually clean the babies?

R: Yes, those that are not very sick and their mothers are not coming, so you just can’t leave them dirty and their ointment is there, you wash them then you change then, you make the cots then you feed them, that’s when your work will be over

I: Is that also in your job description?

R: No, it isn’t there it is just helping. (Support Staff, Hospital 2)

A clear example of acceptance of a need for support (and therefore task-shifting) was seen in hospital 3 where the hospital had negotiated with its administration to have one support worker permanently allocated to the NBU ward. We observed this person undertaking a host of basic care tasks, e.g. cleaning incubators, top tailing and nasogastric tube feeding and also general management of patients.

They [support staff] are more accessible to patients because the nurse is in the station writing Kardex and doing/ handling other issues so the complaints from the patients they take them and they come to tell the nurse ‘patient so and so is requesting for this’…. (NBU nurse, Hospital 3)

Where nurses considered some tasks simple and therefore ‘safe’ to delegate, the important aspects of the logic behind such tasks was often lost to those it was delegated to. For example, clinical staff at Hospital 1 initiated teaching lessons for cleaners about infection control and its importance. While the cleaners ensured everything looked clean, they were often found to be wiping down surfaces with dirty cloths, unaware that surfaces may look clean while harbouring infection.

There are tasks that can be done by such a person [support staff] …, like feeding, changing, top tailing, or even dusting, even something like cleaning an incubator someone who is not skilled can be trained and do all that, we don’t have to have a nurse. Okay, the issues of infection prevention, but you can teach someone to do that…. (Nurse manager Hospital 1)

We found that tasks delegated were not always supervised, even where mentoring of students was expected. From our observations, nurses spend minimal time teaching junior staff and students despite these hospitals being training facilities. Nursing students, however, perceived this as the norm, accepting it as the learning culture in public hospitals:

R1: In comparison, we can say okay in our hospital you know our hospital is a private hospital, so in private, most of the tasks… we don’t do them, the nurses the qualified nurses are the ones who do

I: Tell me what they do

R2: Like the resuscitations, fixing of NG tubes, cannulas and giving some medications

I: And fixing NG tubes?

R1: Yeah, here [public hospitals] you do everything. In NBU we… you just find yourself giving treatment, doing something…. (Nursing students Hospital 2)

Our ethnographic observations of what routinely happened in the wards as described above contrasted with the ‘official position’ of senior nurses offered during interviews. The experts were concerned about safety and levels of competency and were cautious about what ought to be delegated and what ought to remain within the nursing profession (see stakeholders’ views in Additional file 1: Table S1). There was a general agreement that nurses could delegate a task to a student or other carer only if they retained responsibility for such tasks being carried out correctly and for appropriately supervising the student(s). The position of senior nurses not on the frontline of practice, was simply that, nurses had to work harder to comply with professional standards of care, regardless of the pressure they were under.

## Discussion

Our work sought to gain an understanding of how neonatal care is organized and structured [40] on a daily basis and what opportunities exists for task shifting. We believe that the professional cultures, occupational jurisdictions and de facto templates of care of frontline workers will directly influence the success or failure of task-shifting interventions. We have attempted to reveal these contextual factors so that the design of any task-shifting intervention is more considered and hence more likely to succeed.

As there were no explicit job descriptions and standard work guidelines for routine newborn work, the working model of nursing was normatively formed over many years in response to the practical realities of the environment. Hospitals are replete with organizational timetables and schedules [41], but to the nurses, this structuring offered limited guidance on how to organize and delegate certain tasks. Therefore, as a way of coping with task ambiguity, nurses developed de facto routines that provided direction on what they should be doing at any given time (see Fig. 1). However, because of the unpredictable nature of events in these settings [42], the routines would often be disrupted, and nurses would find themselves quickly having to make difficult decisions. In such situations, nurses prioritized nursing tasks based on patient needs using ‘subconscious triage’, which often included delegating and shifting tasks to others.

Delegation of tasks was, however, ad hoc as support staff, students and mothers frequently worked without supervision, and none seemed concerned about undertaking tasks that they were not trained for. This contrasted with findings of an expert meeting conducted as part of our broader programme of work [39] where attendees firmly expressed consensus on tasks that should be formally conducted by nurses and on tasks where delegation might be allowable.

From a policy perspective, our insights suggest that there is potential space for formal task shifting within the everyday routines of neonatal nursing in Kenya. We suggest that careful study of context can help inform task-shifting design. When asked formally about the prospects for task-shifting, the nurses we spoke to had doubts and concerns, but their near continual reliance on organic forms of task shifting suggest that a carefully designed programme could be accepted. Kessler, Heron and Dopson [27] reveal in the United Kingdom health context that post-implementation, health care assistant roles are nearly always viewed very positively by nurses, patients and the new cadres themselves. They do however warn that the specific usefulness of these new human resources is rarely well understood, and hence, they are not strategically deployed despite their obvious potential in addressing HRH needs. Our findings also indicate that considerable work may be required at the policy level, dominated by professional institutions, experts and senior managers, to help bridge the apparent gap in acceptance of task shifting in this clinical arena.

Despite delegating many ‘nursing tasks’ to others, nurses maintained their distinctive role and power by authorizing to whom and how delegation of tasks was orchestrated in their wards. Their authority was mainly drawn from physically spending most of their time in the NBU and being constantly engaged in direct patient care, in contrast with the other more medical cadres. Nurses experiential knowledge of the wards, and what works and does not work, consequently authored the patterns of social organization and culture of work within the ward [43]. These are reinforced in the NBUs by the routines nurses keep. Routines and rituals exist to serve different needs [44, 45], and in our case, a template of routines helped nurses mentally manage tasks within shifts. They offer nurses a sense of control and accomplishment within chaotic work environments characterized by limited resources and staff shortages that make completion of all tasks, as the experts and seniors would have them conducted, an impossibility.

The description of organic practices and local norms established in response to work pressures provides useful guidance for the design of task-shifting initiatives [22]. For instance, we see that nurses prioritize their more technical roles. This inadvertently moves them further away from their traditional roles of providing bedside care and consequently helps delineate potentially acceptable nursing and task-shifting roles [46, 47]. To achieve multi-disciplinary holistic care, it is, however, important that professional role boundaries are negotiable and occupational jurisdictions remain flexible [43, 48], but the starting point for such endeavours is ensuring that roles are defined, that scopes of practice are understood, and that task allocations match capabilities.

Past studies in LMICs have described how lay health workers’ (LHWs) credibility is enhanced through visible ties to the health system through, among other things, visible contact with health professionals through referrals, supervisory visits and involving health professionals in training LHWs [20, 21]. In short, preliminary evidence suggests lay health workers are well-liked by patients. Health systems are however complex, dynamic and political systems, and this is particularly true in Kenya after the recent devolution, which has exacerbated human resources management challenges including disruptions, delays, and discrepancies in health workers’ salaries; resulted in political interference and discrimination in HRH management; and prompted frequent industrial actions by health workers [49]. The working environment of health workers across health facilities in the country has been characterized by fear, anxiety, mass resignations and low health worker morale. Many of these issues would affect a new cadre of health workers as they do the existing cadres. Challenges of health worker shortages and human HRH management in Kenya are chronic and largely remain unaddressed, and it is important that stakeholders do not perceive task shifting as a ‘fix all’ solution.

### Limitations of the study

- The title of this piece refers to ‘Kenyan hospitals’, but we recognize that our subset of urban examples may not address the experience of rural hospitals particularly well. It should be noted that rural hospitals often have even greater struggles in securing staff and so task-shifting may be more attractive to these organizations.
- Further, we recognize that our research was conducted during a period of significant unrest in the Kenyan health system and that the strains we observed may have been exaggerated by this.
- Finally, this study points to the potential space available for task shifting, and the needs such an approach might address, but it does not provide evidence of the likely success of task shifting in addressing major HRH shortfalls

## Conclusion

In summary, our work described a routine template of neonatal nursing work which nurses used to enhance predictability when working in chaotic, resource-limited work environments. We detailed how this model of care deprioritizes less technical or less clinical tasks, which are then left to students, parents or other staff through ‘organic’ task shifting. Such insights are critical when considering more formal task-shifting projects as they help to delineate the tasks most easily shifted, and how best to support and work with busy nurses. While the profound effects of existing health system challenges (e.g. devolution, frequent strikes, delayed salaries and complexities around pay on hospital operations) on human resources management must also be addressed, our findings suggest there is space for a well-designed task-shifting programme that could have a positive and supportive effect on nursing care and on nurses themselves.

## Additional file

Additional file 1: Interview guide. (DOCX 23 kb)

## Abbreviations

GHWA: Global Health Workforce Alliance
HRH: Human resources for health
LHWs: Lay health workers’
LMICs: Low- and middle-income countries
NBUs: Nairobi’s New Born Units
NGT: Naso-gastric tube
OPTIMIZEMNH: Optimizing Health Worker Roles to Improve Access to key Maternal and Newborn Health Interventions
WHO: World Health Organization
