# Supplementary material for: Exploring the space for task shifting to support nursing on neonatal wards in Kenyan public hospitals
Source: Hum Resour Health. 2019 Mar 6;17:18. doi: 10.1186/s12960-019-0352-x (PMC6404312; doi:10.1186/s12960-019-0352-x)
Supplement: Supplementary file 1 — Interview guide. (DOCX 23 kb) [file 12960_2019_352_MOESM1_ESM.docx]

# Additional file 1

## Table S1

| **Task area** | **Recommended person to perform task** | **Persons observed performing task** |
| --- | --- | --- |
| **DRUGS** |  |  |
| Oral drug administration | Nurse | Student nurses often without supervision |
| IV drug administration | Nurse | Student nurses with minimal supervision |
| **FEEDING** |  |  |
| Teaching / counseling on Breast feeding (attachment/suck) | Nurse/nutritionist | Student nurses, mothers, support staff often with minimal supervision |
| Cup feeding | Nurse/mother under supervision | Student nurses, mothers, support staff often with minimal supervision |
| NG tube feeding | Nurse/mother under supervision | Student nurses, mothers often with minimal supervision |
| Charting feed volumes / times | Nurse/mother under supervision | Student nurses, mothers often with minimal supervision |
| **MILK PREPARATION** |  |  |
| Formula making | Nurse/ nutritionist/mother | Student nurses, mothers, support staff often with minimal supervision |
| Measuring volumes for individual babies | Nutritionist | Nurses, student nurses, mothers often with minimal supervision |
| **COUNSELLING** |  |  |
| Supervision of mother during Kangaroo mother care | Nurse/clinician | Nurses and student nurses but seldom done |
| Health education and progress | Nurse | Nurses and student nurses but seldom done |
| Expressing breast Milk | Nurse/ nutritionist/experienced mothers | Nurses (rarely), student nurses and mothers but with minimal supervision |
| Bereavement counselling | Nurse/clinician | Nurses (rarely), social worker (only on request for emotionally difficult cases), student nurses and mothers but seldom done |
| **REGULAR BABY CHECKS** | | |
| Weight | Nurse/clinician | Nurses, nutritionists and student nurses and in some hospitals support staff |
| **VITALS** | | |
| Temperature | Nurse/clinician | Nurses and student nurses (often no supervision) |
| Pulse | Nurse/clinician | Nurses and student nurses (often no supervision) |
| Respiration | Nurse/clinician | Nurses and student nurses (often no supervision) |
| Oxygen saturation | Nurse/clinician | Nurses and student nurses (often no supervision) |

***Table S1 highlighting how official recommendations on persons responsible contrast with practical realities observed during empiric research***

## Study collection tools

## Semi-Structured Interview Guide: Phase 1

### Closed Questions

| Interviewee Code |  |
| --- | --- |
| Organisation |  |
| Position |  |
| Grade |  |
| Age |  |
| Experience in Neonatal (yrs) |  |
| Experience of Nursing (yrs) |  |
| Experience with this org (yrs) |  |

### Open-Ended Questions – Example Questions

Can you explain the history of your organisation?

*<Note any interesting areas and come back to any key points later in interview>*

As a nurse, what are benefits of joining your organisation?

*<Record any overlap with other organisations, step into each area of responsibility/jurisdiction with ‘how do you do that?’. Also record certification and assessment processes.>*

What are the big concerns in nursing in general at this time?

*<Don’t lead at first. After the interviewee is completely finished, introduce other issues identified throughout research>*

Do you have any specific involvement in neonatal nursing? How do these larger issues affect the provision of care in this area?

*<Step into the three core Nairobi hospitals>*

We’re interested in understanding the workload of nurses and if they could be helped to provide better care. We understand that there are fewer nurses that the Kenyan recommendations suggest there should be. What are the effects of this?

<Uses expansion techniques such as saying ‘I don’t understand’ and ‘can you tell me more about that’ in order to explore all the key issues here.>

What is your organisation doing to target these issues?

<Fully record and explore all interventions, efforts and representation the organisation is involved in>

We’d like to explore this area and help generate new ideas about how to improve care. We’re interested in experimenting shifting some of the more simple tasks onto a lower cadre of staff to relieve the pressure on nurses and to allow them to do more clinical work. We’re interested in having your ideas and direction to help explore this idea. Could you help us?

*<Explore any ideas, concerns etc. Take as much time and care as possible, noting concerns and ideas and coming back to them individually>*

Thank the interviewer for their time, explain how we hope to involve them in the further roll-out of the project.

## Semi-Structured Interview Guide: Phase 2

### Closed Questions

| Interviewee Code |  |
| --- | --- |
| Hospital |  |
| Position |  |
| Grade |  |
| Age |  |
| Experience in Neonatal (yrs) |  |
| Experience of Nursing (yrs) |  |
| Experience on this ward |  |
| Membership of Nurse Orgs |  |
| Distance to residence (time, kms) |  |

### Open-Ended Questions – Example Questions

Can you explain what your major tasks were today?

*<<Step through all major tasks, asking for details>>*

Is that a normal shift?

*<<Investigate what ‘normal’ is and what constitutes ‘easy’, ‘busy’, ‘exhausting’ etc. Step into each task and ask how workload and responsibilities are dealt with.>>*

How does that compare to when you started?

*<<Step into variation over time, understandings of the fairness of changes made>>*

Have you been involved in any improvement projects? What was helpful to you as a nurse?

*<<Identify correlations between areas highlighted above as causing difficulty and interventions that helped>>*

Do you have many friends that are also nurses? How is their experience?

*<<Investigate: variation across: grades; public/private; salaries; specialty; experience; social ties etc. >>*

You’re a ** grade, how would changing your position help? What about education? Where do you want your career to go?

*<<List priorities but bracket out pay, bonuses, education after all listed. Concentrate on what is important in terms of nursing identity.*

Are you a member of any organisations, associations, unions? What do they do for you?

*<<General exploration of importance. Step into any particular instances where they’ve assisted the individual and step through as a story>>*

One of the things we’re interested in, is in helping high risk newborns. What can we do to provide them better care?

*<<Seek positive examples: Who is doing it well? How are they doing it? What is good there? >>*

We’re also interested in investigating if your work could be helped if some of the easy tasks were taken care of by someone else. Are there any ‘easy’ tasks that would allow you to spend more time on the critical things?

*<<List any tasks already highlighted as ‘easy’ – ask interviewee to step into easier tasks – work hard to help interviewee identify points of difference: the things that make tasks easy and hard.>>*

If there were a new lower cadre of staff, trained to help nurses in neonatal wards, do you think this would be a good/bad thing (why)?

*<<Do not lead. Very important to let the interviewee express their view based on the consideration of the last questions. If negative, explore reasons why negative and offer potential fixes. If positive, explore objectives raised in other interviews.>>*

Do you want to tell me anything else about neonatal nursing, or your work more generally?

*<<Offer opportunity to discuss any qualms, concerns or questions about the research.>>*

Thank the interviewee for their time, explain the project again, and inform them that they will be made aware of findings.

**Non- participatory Observation Guide**

**Aim:** In an informal manner this method will include listening and watching the activities listed below (guide). While non-participatory in nature, the observations involved delicate probing on what was being observed from the nurses as well as other available staff. The observations allowed the researchers to familiarize themselves with the nurses’ working context and to engage with the participants to build rapport, to break down distinctions between the researcher and participants, and to have an insider perspective

| **TYPE OF ACTIVITY TO BE OBSERVED** | **SOURCE OF OBSERVATION** |
| --- | --- |
| Day to day nursing management of patients | Ward rounds and hand-overs  Routine activities-feeding, monitoring, administering medicines and counselling |
| Supervision, Mentoring and coaching of staff | Ward rounds, handovers and CMEs and informal chats& encounters the ward |
| Documentation duties | Hand-overs, updating the cardex, updating patients’ files clinics |
| Ordering of drugs and supplies | Informal chats and encounters in the ward |
| Staff communication and information exchange | Departmental meetings (may be cadre specific), informal chats& encounters |
| Interaction with senior staff | Daily report handover and Informal chats& encounters |
| Interaction with peers | Ward rounds, hand-over, informal chats& encounters |
| Interaction with juniors | Ward rounds, hand-over, informal chats& encounters |

Other than the above, it will be useful to observe and documenting the following

- Description of the facility
- Organogram of the hospital
- Overall patient volume
- Neonatal patient volume
- Organization of services
- Lay out of service points
- Patient flow (from out-patient to in-patient)
- Organization of shifts within the neonatal ward
- Main procedures in the neonatal ward
- Human Resource Capacity
- Number of staff within the facility
- Neonatal nurses within the facility
- Number of nurses per shift in the neonatal ward
